# Supplementary material for: Surveying the global landscape of post-transcriptional regulators
Source: Nat Struct Mol Biol. 2023 May 25;30(6):740–52. doi: 10.1038/s41594-023-00999-5 (PMC10279529; doi:10.1038/s41594-023-00999-5)
Supplement: Supplementary file 1 — Supplementary Tables 1–3. [file 41594_2023_999_MOESM1_ESM.pdf]

nature structural & molecular biology

Article

https://doi.org/10.1038/s41594-023-00999-5

Surveying the global landscape of post-transcriptional regulators

In the format provided by the authors and unedited

Tables

Supplementary Table 1: Plasmids used in this study.

Plasmid name	Purpose	Expression cassette	Source
pKS038	Sgn1 tethering vector	pPGK1::SGN1-λN:-SpHis5::tADH1 ARS/CEN	This study
pKS109	Halo control tethering vector	pPGK1::Halo-λN-SpHis5::tADH1 ARS/CEN	This study
pKS132	Library in-frame selection vector	pPGK1::λN-P2A-SpHis5::tAgTEF ARS/CEN	This study
pKS137	Library tethering vector	pPGK1::λN-T2A*-1xFLAG-BFP-NES::tADH1 pAgTEF::SpHIS5::tAgTEF ARS/CEN	This study
pKS151	Ded1(14-178) tethering vector	pPGK1::Ded1(14-178)-λN-T2A*-1xFLAG-BFP-NES::tADH1 pAgTEF::SpHIS5::tAgTEF ARS/CEN	This study
pKS152	Ebs1(691-876) tethering vector	pPGK1::Ebs1(691-876)-λN-T2A*-1xFLAG-BFP-NES::tADH1 pAgTEF::SpHIS5::tAgTEF ARS/CEN	This study
pKS153	Hsp26(1-182) tethering vector	pPGK1::Hsp26(1-182)-λN-T2A*-1xFLAG-BFP-NES::tADH1 pAgTEF::SpHIS5::tAgTEF ARS/CEN	This study
pKS154	Ngr1(474-626) tethering vector	pPGK1::Ngr1(474-626)-λN-T2A*-1xFLAG-BFP-NES::tADH1 pAgTEF::SpHIS5::tAgTEF ARS/CEN	This study
pKS155	Tif35(115-239) tethering vector	pPGK1::Tif35(115-239)-λN-T2A*-1xFLAG-BFP-NES::tADH1 pAgTEF::SpHIS5::tAgTEF ARS/CEN	This study
pKS156	Gta1(603-767) tethering vector	pPGK1::Gta1(603-767)-λN-T2A*-1xFLAG-BFP-NES::tADH1 pAgTEF::SpHIS5::tAgTEF ARS/CEN	This study
pKS160	Her1(1025-1142) tethering vector	pPGK1::Her1(1025-1142)-λN-T2A*-1xFLAG-BFP-NES::tADH1 pAgTEF::SpHIS5::tAgTEF ARS/CEN	This study
pKS171	Sro9(14-151) tethering vector	pPGK1::Sro9(14-151)-λN-T2A*-1xFLAG-BFP-NES::tADH1 pAgTEF::SpHIS5::tAgTEF ARS/CEN	This study
pKS173	Sbp1(1-108) tethering vector	pPGK1::Sbp1(1-108)-λN-T2A*-1xFLAG-BFP-NES::tADH1 pAgTEF::SpHIS5::tAgTEF ARS/CEN	This study
pKS174	Smy2(65-232) tethering vector	pPGK1::Smy2(65-232)-λN-T2A*-1xFLAG-BFP-NES::tADH1 pAgTEF::SpHIS5::tAgTEF ARS/CEN	This study

pKS176	Jsn1(144-295) tethering vector	pPGK1::Jsn1(144-295)-λN-T2A*-1xFLAG-BFP-NES::tADH1 pAgTEF::SpHIS5::tAgTEF ARS/CEN	This study
pKS177	Cdc48 tethering vector	pPGK1::CDC48-λN-T2A*-1xFLAG-BFP-NES::tADH1 pAgTEF::SpHIS5::tAgTEF ARS/CEN	This study
pKS179	Yap1801 tethering vector	pPGK1::YAP1801-λN-T2A*-1xFLAG-BFP-NES::tADH1 pAgTEF::SpHIS5::tAgTEF ARS/CEN	This study
pKS180	Sbp1 tethering vector	pPGK1::SBP1-λN-T2A*-1xFLAG-BFP-NES::tADH1 pAgTEF::SpHIS5::tAgTEF ARS/CEN	This study
pKS182	Sro9 tethering vector	pPGK1::SRO9-λN-T2A*-1xFLAG-BFP-NES::tADH1 pAgTEF::SpHIS5::tAgTEF ARS/CEN	This study
pKS183	Gta1 tethering vector	pPGK1::GTA1-λN-T2A*-1xFLAG-BFP-NES::tADH1 pAgTEF::SpHIS5::tAgTEF ARS/CEN	This study
pKS190	*Gta1Δ603-767* tethering vector	pPGK1::Gta1Δ603-767-λN-T2A*-1xFLAG-BFP-NES::tADH1 pAgTEF::SpHIS5::tAgTEF ARS/CEN	This study
pKS192	Sro9-3xFLAG tethering vector	pPGK1::SRO9-3xFLAG-λN-T2A*-1xFLAG-BFP-NES::tADH1 pAgTEF::SpHIS5::tAgTEF ARS/CEN	This study
pKS193	Sro9(1-151)-3xFLAG tethering vector	pPGK1::SRO9(1-151)-3xFLAG-λN-T2A*-1xFLAG-BFP-NES::tADH1 pAgTEF::SpHIS5::tAgTEF ARS/CEN	This study
pKS194	Sro9(1-251)-3xFLAG tethering vector	pPGK1::SRO9(1-251)-3xFLAG-λN-T2A*-1xFLAG-BFP-NES::tADH1 pAgTEF::SpHIS5::tAgTEF ARS/CEN	This study
pKS196	Halo-3xFLAG tethering vector	pPGK1::Halo-3xFLAG-λN-T2A*-1xFLAG-BFP-NES::tADH1 pAgTEF::SpHIS5::tAgTEF ARS/CEN	This study
pKS207	Gta1 inducible vector	pGAL1::GTA1-λN-T2A*-1xFLAG-BFP-NES::tADH1	This study
pKS208	*Gta1Δ603-767* inducible vector	pGAL1::Gta1Δ603-767-λN-T2A*-1xFLAG-BFP-NES::tADH1	This study
pKS232	Halo inducible vector	pGAL1::Halo-λN-T2A*-1xFLAG-BFP-NES::tADH1	This study
pNTI282	YFP-boxB vector	pCMV::eGFP::5xboxB::poly(A) BGH	This study
pNTI473	RFP-PP7 vector	pPGK1::mCherry::3xPP7::tADH1	This study
pHES795	ZIF268 synthetic transcription factor	Zif268 DBD-hPR LBD-MSN2 AD	[91]

	vector		
pHES840	pGAL1 inducible promoter vector	pGAL1::YFP	[91]
pCfB2189	Leu+ vector	KILEU2 at integration site X-3	[92]
pCfB2225	Kan+ vector	KanMX at integration site XII-2	[92]
pCfB2337	Hygromycin+ vector	HphMX at integration site XII-5	[92]
pNTI114	YFP::boxB integration vector	CCR5_3'HR:P_CMV::eGFP::polyA_BGH:CCR5_5'HR	This Study
pNTI252	RFP::boxB integration vector	pFA6a P(PGKI)::mCherry:boxB::T(ADH1) CaURA3 URA3int	This Study
pNTI473	RFP::PP7 integration vector	pFA6a P(PGK1)::mCherry:pp7(x3):T(ADH1) CaURA3 URA3int	This Study
pNTI476	YFP::PP7 vector	pFA6a P(PGK1)::YFP:pp7:T(ADH1) CaURA3 URA3int	This Study
pNTI729	ZIF268 synthetic transcription factor integratable vector	pADH1::ZIF268::C.albicans tADH1 at site XII-6	[93]

Supplementary Table 2: Strains used in this study.

Name	Genotype	Purpose	Source
NIY106	MATα his3Δ1 leu2Δ0 lys2Δ0 MET15 pPGKI::mCherry:boxB:CaUra3	RFP::boxB haploid strain	This study
NIY111	MATa his3Δ1 leu2Δ LYS2 met15Δ ura3Δ0	BY4741 MATa WT yeast	ThermoFisher
NIY112	MATα his3Δ leu2Δ0 lys2Δ0 MET15 ura3Δ0	BY4742 MATα WT yeast	ThermoFisher
NIY114	MATa his3Δ1 leu2Δ LYS2 met15Δ pPGKI::YFP:boxB:CaUra3	YFP::boxB haploid strain	This study
NIY286	MATα his3Δ1 leu2Δ0 lys2Δ0 MET15 pPGKI::mCherry:PP7:CaUra3	RFP::PP7 haploid strain	This study

NIY287	MATa his3Δ1 leu2Δ LYS2 met15Δ pPGKI::YFP:pp7:CaUra3	YFP::PP7 haploid strain	This Study
NIY289	MATα/MATa his3Δ1/his3Δ1 leu2Δ0/leu2Δ0 lys2Δ0/LYS2 MET15/met15Δ pPGKI::mCherry:boxB:CaUra3/pPGKI::YFP:pp7:CaUra3	YFP::PP7/RFP::boxB dual reporter strain	This Study
NIY293	MATα/MATa his3Δ1/his3Δ1 leu2Δ0/leu2Δ0 lys2Δ0/LYS2 MET15/met15Δ pPGKI::mCherry:pp7:CaUra3/pPGKI::YFP:boxB:CaUra3	YFP::boxB/RFP::PP7 dual reporter strain	This study
yKS090	MATα/MATa his3Δ1/his3Δ1 leu2Δ0/leu2Δ0 lys2Δ0/LYS2 MET15/met15Δ HygR pPGKI::mCherry:pp7:CaUra3/pPGKI::YFP:boxB:CaUra3 pADH1::ZIF268::tADH1	Dual reporter strain NIY293 with ZIF268 synthetic transcription factor integrated at XII-5 site	This study
yKS092	MATα/MATa his3Δ1/his3Δ1 LEU2/leu2Δ0 lys2Δ0/LYS2 MET15/met15Δ *ubx2Δ*/UBX2 KanR pPGKI::mCherry:pp7:CaUra3/pPGKI::YFP:boxB:CaUra3	Dual reporter with 1 copy *ubx2Δ*, 1 copy UXB2	This Study
yKS093	MATα/MATa his3Δ1/his3Δ1 LEU2/leu2Δ0 lys2Δ0/LYS2 MET15/met15Δ *ubx2Δ/ubx2Δ* KanR pPGKI::mCherry:pp7:CaUra3/pPGKI::YFP:boxB:CaUra3	Dual reporter *ubx2Δ* strain	This study
yKS094	MATα/MATa his3Δ1/his3Δ1 LEU2/leu2Δ0 lys2Δ0/LYS2 MET15/met15Δ *ubx2Δ/ubx2Δubx* KanR pPGKI::mCherry:pp7:CaUra3/pPGKI::YFP:boxB:CaUra3	Dual reporter *ubx2Δc* (no UBX domain) strain	This Study

Supplementary Table 3: Oligonucleotides used in this study.

**Name**	**Purpose**	**Sequence**	**Source**
NI-AM-163	*mCherry* qPCR	CATGGTCTTCTTCTGCATTACG	This study
NI-AM-164	*mCherry* qPCR	GACTACTTGAAGCTGTCCTTC	This study
NI-AM-161	*Citrine* qPCR	GTCAGCCATGATGTAAACATTGTG	This study

NI-AM-162	*Citrine* qPCR	GACGGTAACTACAAGACCAGA	This study
KS917	*GTA1* qPCR	CCAGGAGGACTTACACGATGTG	This study
KS918	*GTA1* qPCR	GATATTGGATTTCAAGGATTTCCGC	This study
KS765	*UBC6* qPCR	CCAGTGATGAAGCCACGACAGG	This study
NI-AM-229	*UBC6* qPCR	AAAGGGTCTTCTGTTTCATCACCTGTATTTGC	This study
KS877	*ubx2Δ::kanMX*	CCAGAAGAGATGAAGGTGATATATAGGTAGACAGtaggtctagagatctgtttagcttgc	This study
KS878	*ubx2Δ::kanMX*	TTGTACGCGTTTGTCGTTTTTAACGATATGCTATTTTAattaagggttctcgagagctcg	This study
KS879	*ubx2Δ::kanMX*	GGGCTTCGAGCGCGACAATTGGTGGGTGGTGGCCCAGAAGAGATGAAGGTGATATATAGG	This study
KS880	*ubx2Δ::kanMX*	ACGAAAAATGAAAAAAAAGTAAACTCCAGAAACTCTTTGTACGCGTTTGTCGTTTTTAAC	This study
KS881	*ubx2Δ::KlLEU2*	CGATAGAAGTATGTAATAGCTTTCATAGTGTAATCGAAGGATCCGCAGGCTAACCGGAAC	This study
KS882	*ubx2Δ::KlLEU2*	ATTTGGCCATAGCTGGGGCACTTGGTCCACCGAACCATTCAAATTGAACAGAGAAGTCAG	This study

KS883	*ubx2Δ::KlLEU2*	AGACAGTGTATTTTGTAGCAGCAGGTATTACGATAGAAGTATGTAATAGCTTTCATAGTG	This study
KS884	*ubx2Δ::KlLEU2*	TCTTCATCTTCTTCATCCAGTGCTTCCACTAGAAGACTGCCATTTGGCCATAGCTGGGGC	This study
KS838	*ubx2Δc::KlLEU2*	CACGCTCAGGATTTGAAATTAAACTCAACTGCTCGGTAAGGCGCGCCACTTCTAAATAAG	This study
KS839	*ubx2Δc::KlLEU2*	CTCATCGATGCAAGCCTTTAACCATTTTAATTGACCATTCAAATTGAACAGAGAAGTCAG	This study
KS840	*ubx2Δc::KlLEU2*	AATCAAAGCTATAGAAAAGGAAAAGAGCTTGAAACACGCTCAGGATTTGAAATTAAACTC	This study
KS841	*ubx2Δc::KlLEU2*	GAGTAGCTTGCTTCCCCGTTGTCTCGAACGGCTGTATCTCATCGATGCAAGCCTTTAACC	This study
KS842	*ubx2Δc::KlLEU2* check	CGTGGTGGAAAAGTACACTCCAG	This study
KS843	*ubx2Δc::KlLEU2* check	CCTGAGAAAGCAACCTGACCTAC	This study
KS844	*ubx2Δc::KlLEU2* check	CCGCGAGGTATATGTGACACCC	This study
