# Supplementary material for: Surveying the global landscape of post-transcriptional regulators
Source: Nat Struct Mol Biol. 2023 May 25;30(6):740–52. doi: 10.1038/s41594-023-00999-5 (PMC10279529; doi:10.1038/s41594-023-00999-5)
Supplement: Supplementary file 2 — Reporting Summary [file 41594_2023_999_MOESM2_ESM.pdf]

nature portfolio

Corresponding author(s): Nicholas Ingolia

Last updated by author(s): 11/18/2022

Reporting Summary

Nature Portfolio wishes to improve the reproducibility of the work that we publish. This form provides structure for consistency and transparency in reporting. For further information on Nature Portfolio policies, see our Editorial Policies and the Editorial Policy Checklist.

Statistics

For all statistical analyses, confirm that the following items are present in the figure legend, table legend, main text, or Methods section.

n/a	Confirmed	
☐	☒	The exact sample size (*n*) for each experimental group/condition, given as a discrete number and unit of measurement
☐	☒	A statement on whether measurements were taken from distinct samples or whether the same sample was measured repeatedly
☐	☒	The statistical test(s) used AND whether they are one- or two-sided<br>*Only common tests should be described solely by name; describe more complex techniques in the Methods section.*
☒	☐	A description of all covariates tested
☐	☒	A description of any assumptions or corrections, such as tests of normality and adjustment for multiple comparisons
☐	☒	A full description of the statistical parameters including central tendency (e.g. means) or other basic estimates (e.g. regression coefficient) AND variation (e.g. standard deviation) or associated estimates of uncertainty (e.g. confidence intervals)
☐	☒	For null hypothesis testing, the test statistic (e.g. *F*, *t*, *r*) with confidence intervals, effect sizes, degrees of freedom and *P* value noted<br>*Give P values as exact values whenever suitable.*
☒	☐	For Bayesian analysis, information on the choice of priors and Markov chain Monte Carlo settings
☒	☐	For hierarchical and complex designs, identification of the appropriate level for tests and full reporting of outcomes
☐	☒	Estimates of effect sizes (e.g. Cohen's *d*, Pearson's *r*), indicating how they were calculated

Our web collection on statistics for biologists contains articles on many of the points above.

Software and code

Policy information about availability of computer code

Data collection: BD FACSDiva Software Version 6.2; Leica Application Suite v4.8.0

Data analysis:
ImageJ 1.53t
cutadapt version 1.16
HISAT2 version 2.1.0
bedtools v2.25.0
Custom and open source software used to analyze data in this study are provided in Zenodo at https://zenodo.org/record/6496503

For manuscripts utilizing custom algorithms or software that are central to the research but not yet described in published literature, software must be made available to editors and reviewers. We strongly encourage code deposition in a community repository (e.g. GitHub). See the Nature Portfolio guidelines for submitting code & software for further information.

Data

Policy information about availability of data

All manuscripts must include a data availability statement. This statement should provide the following information, where applicable:

- Accession codes, unique identifiers, or web links for publicly available datasets
- A description of any restrictions on data availability
- For clinical datasets or third party data, please ensure that the statement adheres to our policy

High-throughput sequencing data has been deposited with the NCBI Short Read Archive. Long-read sequencing linking tethering constructs and barcodes is available at SRR10355648, and short-read sequencing quantifying the barcodes is available at SRR10353306 through SRR10353315, as described below.

Accession Title
SRR10355648 Tethering construct library
SRR10353306 Tethering construct barcode, unsorted, replicate 2
SRR10353307 Tethering construct barcode, far-right sort, replicate 2
SRR10353308 Tethering construct barcode, near-right sort, replicate 2
SRR10353309 Tethering construct barcode, near-left sort, replicate 2
SRR10353310 Tethering construct barcode, far-left sort, replicate 2
SRR10353311 Tethering construct barcode, unsorted, replicate 1
SRR10353312 Tethering construct barcode, far-right sort, replicate 1
SRR10353313 Tethering construct barcode, near-right sort, replicate 1
SRR10353314 Tethering construct barcode, near-left sort, replicate 1
SRR10353315 Tethering construct barcode, far-left sort, replicate 1
Publically available data sets used here include
S. cerevisiae proteome and Pfam domain annotations from InterPro proteome UP000002311
S. cerevisiae genome annotations from
http://sgd-archive.yeastgenome.org/sequence/S288C_reference/genome_releases/S288C_reference_genome_R64-2-1_20150113.tgz
BioGRID data from https://downloads.thebiogrid.org/Download/BioGRID/Release-Archive/BIOGRID-4.3.195/BIOGRID-MV-Physical-4.3.195.tab3.zip

Field-specific reporting

Please select the one below that is the best fit for your research. If you are not sure, read the appropriate sections before making your selection.

☒ Life sciences ☐ Behavioural & social sciences ☐ Ecological, evolutionary & environmental sciences

For a reference copy of the document with all sections, see nature.com/documents/nr-reporting-summary-flat.pdf

Life sciences study design

All studies must disclose on these points even when the disclosure is negative.

Sample size	No sample size calculations were performed, and sample sizes were based on previous studies (Muller et al., 2020; Reynaud et al., 2021). Experiments were carried out with biological duplicates or triplicates as described in the text, following approaches commonly accepted for high-throughput genome-wide experiments.
Data exclusions	No data were excluded.
Replication	Experiments were conducted using biological duplicate or triplicate samples as indicated and all replication was successful.
Randomization	Randomization was not relevant to experimental designs in this study: no prospective assignment was performed, control and experimental samples were processed in parallel, and data acquisition and analysis were automated and applied uniformly across all samples.
Blinding	Blinding was not relevant to this study: observer bias is not relevant because data acquisition and analysis are automated and applied uniformly for all samples.

Reporting for specific materials, systems and methods

We require information from authors about some types of materials, experimental systems and methods used in many studies. Here, indicate whether each material, system or method listed is relevant to your study. If you are not sure if a list item applies to your research, read the appropriate section before selecting a response.

Materials & experimental systems

n/a	Involved in the study	
☐	☒	Antibodies
☒	☐	Eukaryotic cell lines
☒	☐	Palaeontology and archaeology
☒	☐	Animals and other organisms
☒	☐	Human research participants
☒	☐	Clinical data
☒	☐	Dual use research of concern

Methods

n/a	Involved in the study	
☒	☐	ChIP-seq
☐	☒	Flow cytometry
☒	☐	MRI-based neuroimaging

Antibodies

Antibodies used	Cell Signaling Technology 2368S DYKDDDDK Tag Antibody (Binds to same epitope as Sigma's Anti-FLAG(R) M2 Antibody) (lot 12)<br>α-Pab1 Antibodies-Online ABIN1580454 (clone 1G1)

	anti-rabbit IgG, HRP-linked (Cell Signaling Technology 7074S, lot 31)<br>anti-mouse IgG, HRP-linked (Cytiva NA931-1ML)
Validation	FLAG/DYKDDDDK epitope tag is widely used to recognize epitope-tagged transgenes in S. cerevisiae (Liu et al., Nat Commun 12: 57 (2021); Sun et al., Cell Reports 36(12): 109717 (2021)). We validate the specificity of this tag by detecting four distinct transgenes at their respective, predicted sizes (Extended Data Fig. 7a).<br>Pab1 antibody is a monoclonal antibody (clone 1G1) previously shown to recognize S. cerevisiae Pab1 by mRNA binding in RNA Interactome Capture that can be competed with free poly-(A) RNA (Matia-Gonzalez et al., STAR Protoc 2021).

Flow Cytometry

Plots

Confirm that:

☒ The axis labels state the marker and fluorochrome used (e.g. CD4-FITC).

☒ The axis scales are clearly visible. Include numbers along axes only for bottom left plot of group (a 'group' is an analysis of identical markers).

☒ All plots are contour plots with outliers or pseudocolor plots.

☒ A numerical value for number of cells or percentage (with statistics) is provided.

Methodology

Sample preparation	Budding yeast were grown in batch culture or turbidostat in mid-exponential growth.<br>Flow cytometry used cells fixed for 30 minutes in 4% PFA<br>Cell sorting used live yeast in 1x PBS<br>All flow cytometry experiments measured genetically encoded fluorescent proteins expressed transgenically
Instrument	BD LSR Fortessa X20 and Aria Fusion sorter
Software	BD FACSDiva Software Version 6.2
Cell population abundance	Fluorescence measurements for 50,000 cells were collected per sample, and gates were drawn to include populations of the ~25% cells with modal forward- and side-scatter
Gating strategy	Fluorescence measurements for 50,000 cells were collected per sample, and gates were drawn to include populations of the ~25% cells with modal forward- and side-scatter<br>Fluorescence activated cell sorting was performed with an Aria Fusion sorter by gating four equal-sized populations based on the ratio of FITC and PE-TexRed emission. Approximately two million cells were sorted into each gate. The sort was performed with two technical replicate libraries from the same library transformation.<br>Gating strategy is exemplified in Extended Data Fig. 3b and 3d

☒ Tick this box to confirm that a figure exemplifying the gating strategy is provided in the Supplementary Information.
