# Supplementary material for: Surveying the global landscape of post-transcriptional regulators
Source: Nat Struct Mol Biol. 2023 May 25;30(6):740–52. doi: 10.1038/s41594-023-00999-5 (PMC10279529; doi:10.1038/s41594-023-00999-5)
Supplement: Source Data Extended Data Fig. 7 — Unprocessed western blots. [file 41594_2023_999_MOESM9_ESM.pdf]

Extended Data Fig. 7b

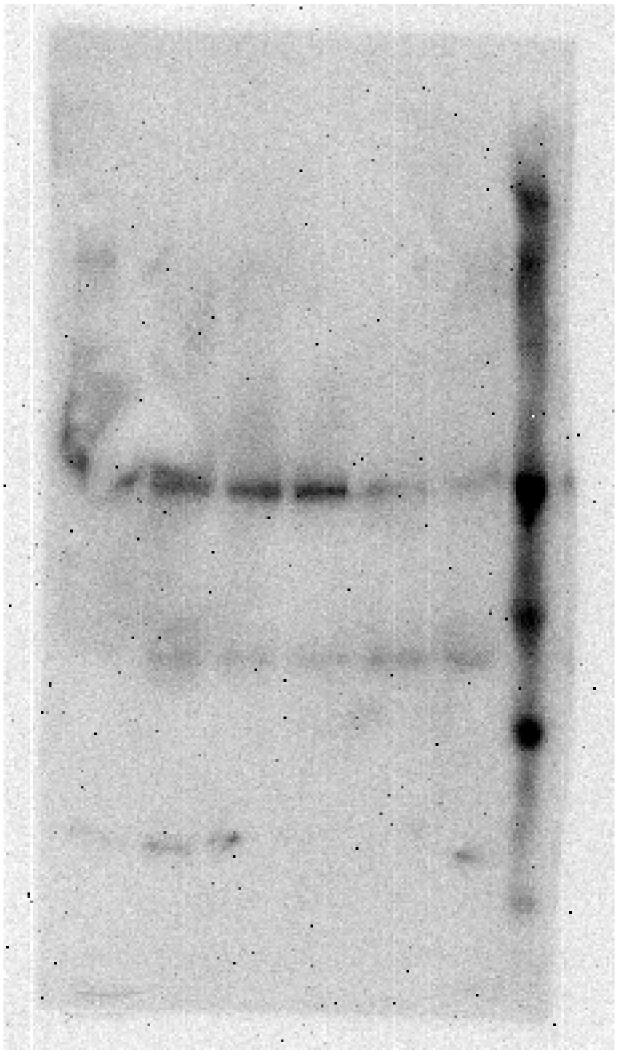

Sample order:
Halo control 2
Halo control 1
Sro9 full-length 2
Sro9 full-length 1
Sro9(1-151) 2
Sro9(1-151) 1

Pab1 expected size: 64 kDa

Extended Data Fig. 7b

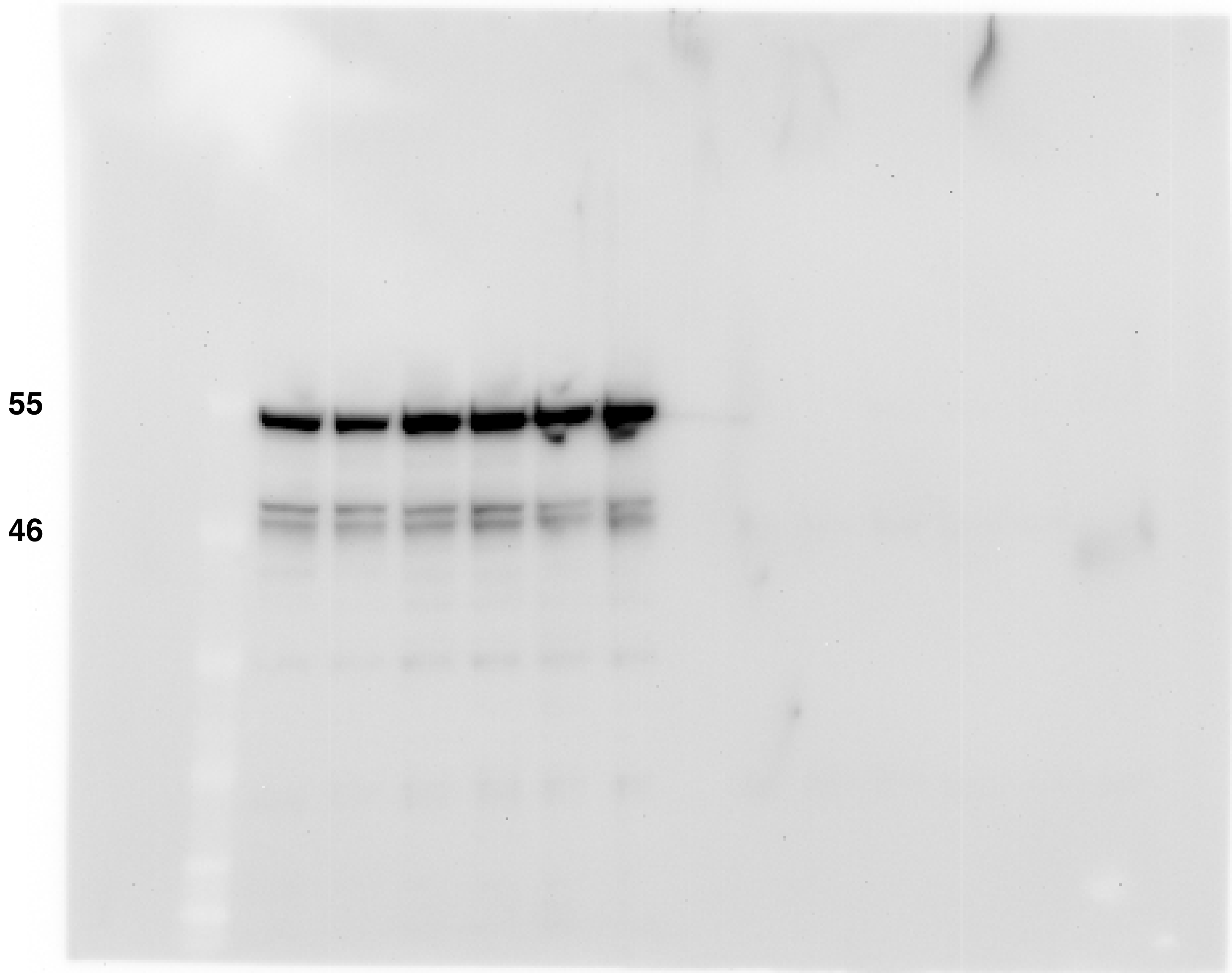

Hxk2 expected size: 54 kDa

Extended Data Fig. 7a

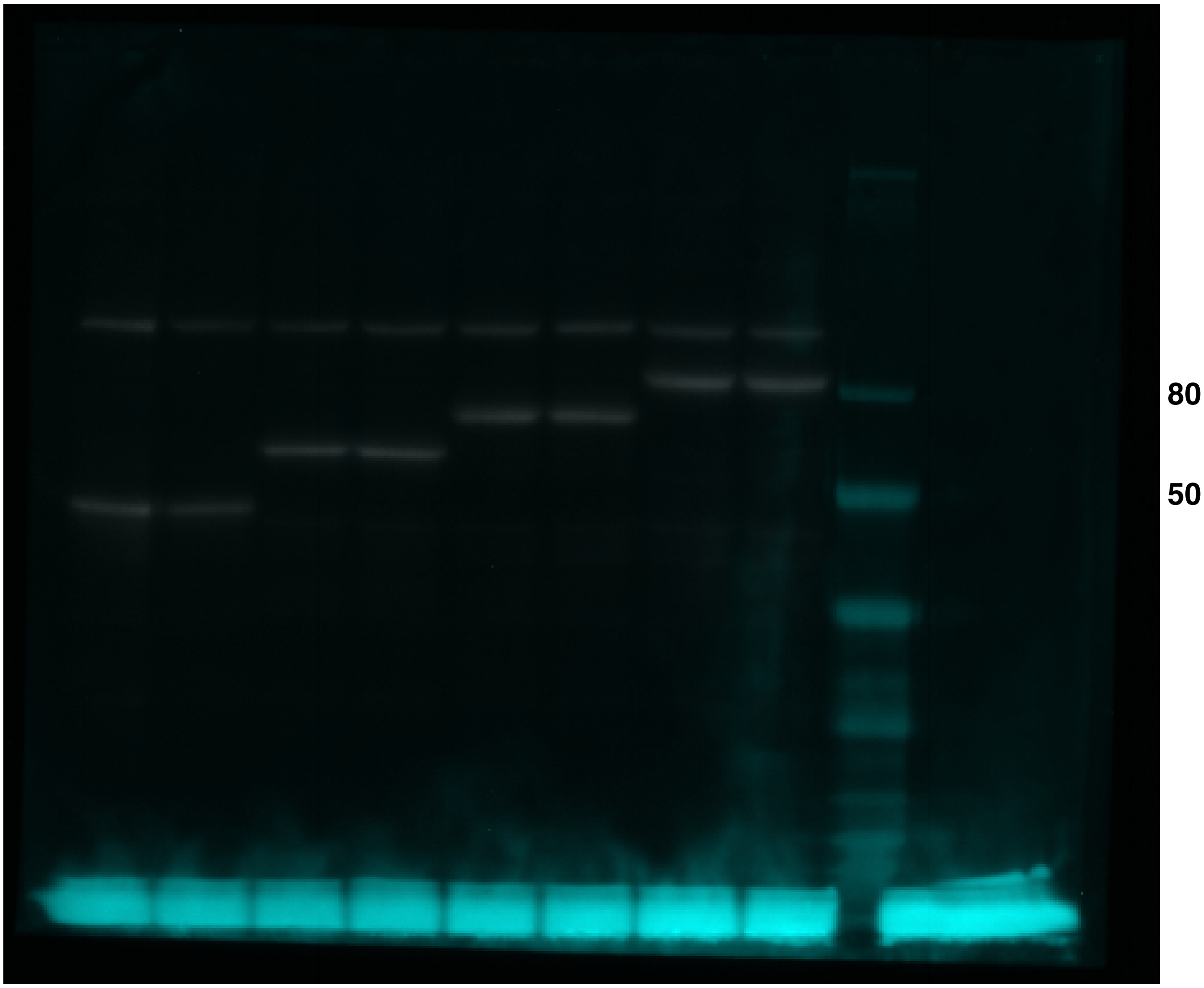

Gel order:

Sro9-FLAG-BFP rep 1: 84 kDa
Sro9-FLAG-BFP rep 2: 84 kDa
Sro9(1-251)-FLAG-BFP rep 1: 64 kDa
Sro9(1-251)-FLAG-BFP rep 2: 64 kDa
Sro9(252-434)-FLAG-BFP rep 1: 56 kDa
Sro9(252-434)-FLAG-BFP rep 2: 56 kDa
Sro9(1-151)-FLAG-BFP rep 1: 51 kDa
Sro9(1-151)-FLAG-BFP rep 2: 51 kDa
